# Physiological Responses and Fatigue during a Repeated Shuttle-Sprint Running Test in Adolescent Schoolchildren: A Comparison between Sexes and Fatigue Calculation Methods

**DOI:** 10.3390/children10061041

**Published:** 2023-06-09

**Authors:** Athanasios Tsoukos, Gregory C. Bogdanis

**Affiliations:** School of Physical Education and Sport Science, National & Kapodistrian University of Athens, 17237 Athens, Greece; atsoukos@phed.uoa.gr

**Keywords:** repeated sprint ability, youth, lactate, performance

## Abstract

We examined physiological responses and fatigue in adolescent boys and girls during a repeated shuttle-sprint running test in a school setting. We also compared three calculation methods to assess fatigue during repeated sprints: the fatigue index (FI), the percent sprint decrement (Sdec), and the slope of performance decrement (SlopeD). Twenty-six adolescent students (10 girls and 16 boys, age: 15.3 ± 0.5 y) performed six 30 m sprints with a 180° change of direction at 15 m, interspersed with 10 s of recovery. Blood lactate (BL), heart rate (HR) and countermovement jump performance (CMJ) were measured before and after the sprint test. Boys achieved significantly faster sprint times (11.7% to 14.8% faster than girls in all sprints, *p* < 0.01), and had higher post-test BL compared with girls (14.0 ± 2.9 vs. 11.3 ± 3.2 mmol/L; *p* = 0.02; d = 0.93). However, all fatigue indices (FI, Sdec and SlopeD) were similar in boys and girls (FI = 11.5 *±* 5.0 vs. 11.5 *±* 5.0; SlopeD = 10.6 *±* 4.8 vs. 14.5 *±* 5.1; Sdec = 5.6 *±* 2.2 vs. 7.3 *±* 2.2, in boys and girls, respectively, *p* > 0.05 for all) and were highly correlated with each other (r = 0.86 to 0.97, *p* < 0.01). CMJ was higher in boys (*p* < 0.05), but CMJ performance dropped similarly from pre- to post-test in boys and girls (13.7 ± 7.0%, *p* < 0.01). In conclusion, neuromuscular fatigue during and after repeated shuttle-sprint running is similar in boys and girls, despite the faster sprint times, higher CMJ and higher BL in boys. SlopeD may be used as an alternative index to quantify fatigue during repeated shuttle-sprinting, as it takes into account performance in all sprints and may provide an average sprint-by-sprint performance drop.

## 1. Introduction

Repeated sprint ability (RSA) is the ability to maintain performance during repeated maximal running efforts with short recovery intervals [1,2]. RSA is important in team sports that are popular among adolescents, such as soccer, basketball, and volleyball [3,4,5], while repeated sprints have been used in a school setting in the form of small-sided games to improve cardiometabolic health and cognitive performance [6,7,8]. Moreover, high intensity activities in the form of repeated short efforts are characteristic of the spontaneous physical activity of children [8]. Therefore, RSA is important for both recreational activities and health promotion in children and adolescents.

Both laboratory and field tests have been employed to determine the level of RSA [9,10,11,12,13,14]. Field tests have high ecological validity and may include linear-only sprinting, such as the running anaerobic sprint test, or may involve changes of direction (commonly by 180°) during each sprint bout [9,13,15,16]. The sprint distance in these tests may range from 15 to 40 m and performance has been correlated with anaerobic power [17], speed at the anaerobic threshold [18], maximal oxygen uptake (VO2max) [19], and neuromuscular performance [9,16,20]. There is evidence showing that RSA performance is independent of whether a linear (e.g., 6 × 25 m) or a change of direction sprint is used (e.g., 6 × [2 × 12.5 m]), implying that RSA is a generic quality which may be assessed with either protocol [21]. The degree of performance drop during RSA tests has also been correlated with neuromuscular fatigue measured by countermovement jump performance (CMJ) [22,23], as well as with increases in blood lactate [22]. During RSA tests, blood lactate rises to 7–15 mmol∙L^−1^ depending on the total distance covered, sex and fitness level of the participants [21,24,25,26,27]. In addition, during RSA tests, heart rate increases near maximum values (178–200 bpm) [18,26,28] and biomechanical parameters, such as stride length and vertical stiffness, are reduced [29].

Fatigue during sprinting is different in childhood and adolescence than in adulthood, with prepubertal children showing little or no fatigue, low glycolytic metabolism, and higher aerobic contribution to energy supply, while puberty reverses this metabolic and fatigue profile towards the adult patterns [30,31]. However, there are limited results regarding the differences in fatigue and physiological responses to repeated intense exercise in pubertal boys and girls [32,33]. In young adult athletic populations performing a repeated sprints test, there are studies showing either a higher fatigue index (FI) in males compared with females [27], or similar FI in the two sexes [28]. Furthermore, blood lactate concentration has been found either to increase equally between sexes [13], or to be higher in males compared with females [28].

Due to the increasing use of high intensity repeated sprint protocols for health promotion in schoolchildren [34], and because of the importance of RSA during recreational sports in children and adolescents [35,36], research is still needed to determine the level of fatigue during repeated sprints and the possible differences between boys and girls who are not training systematically. This would provide information for optimizing the design of repeated sprint training programs aiming to improve performance and health in boys and girls. Thus, the purpose of the present study was to examine and compare fatigue and physiological responses during a repeated shuttle-sprint running test in boys and girls in a school setting. A secondary purpose of this study was to compare three calculation methods to assess fatigue: the fatigue index (FI), the percent sprint decrement (Sdec), and the slope of performance decrement (SlopeD) [37].

## 2. Materials and Methods

### 2.1. Subjects

Twenty-six (n = 26) adolescent 4th grade high school students consisting of 10 girls (age: 15.3 ± 0.5 y, height: 1.66 ± 0.08 m, body mass: 58.5 ± 9.6 kg) and 16 boys (age: 15.4 ± 0.5 y, height: 1.73 ± 0.06 m, body mass: 69.2 ± 7.2 kg) took part in the study. The participants were free of musculoskeletal injuries for at least 1 year prior to the study, were not taking any drugs or nutritional supplements and regularly participated in the physical education lessons (3 × 45 min per week), but not in organized systematic training.

The parents of the students were informed in writing about the aim of the study and the possible risks involved, and they signed an informed consent form. The study was approved by the institutional review board at the School of Physical Education and Sport Science in the National and Kapodistrian University of Athens (Approval no. 1282/19-5-2021). All procedures were in accordance with the Code of Ethics of the World Medical Association (Helsinki declaration of 1964, as revised in 2013).

### 2.2. Experimental Design

This study took place in a high-school setting. All measurements were performed on an indoor non-slippery court and students were instructed to wear sports clothes and shoes. On the first visit, anthropometric measurements were performed (body height and mass, sum of four skinfolds) and the participants were familiarized with counter-movement jump (CMJ) and the shuttle repeated sprint-running test. On the second visit, one week later, the participants performed the main trial which included six 30 m sprints with a 180° change of direction at 15 m. The passive recovery interval between sprints was 10 s. Sprint performance time was measured by a timing gate which was set at the start, which was also the end of each sprint. A standardized warm-up was performed 5 min before the repeated sprints test. Blood lactate countermovement jump (CMJ) and heart rate were measured after the warm-up, and again after the main test.

### 2.3. Measurements

#### 2.3.1. Anthropometric Measurements

Body height was measured by a stadiometer (Charder HM-200P Portstad, Charder Electronic Co., Ltd., Taichung City, Taiwan) to the nearest 0.01 cm. A digital scale (TBF-300A Body Composition Analyzer-Tanita, Tanita Corporation, Tokyo, Japan) was used to measure body mass to the nearest 0.1 kg. Body fat percentage was estimated from the sum of four skinfold thicknesses (biceps, triceps, subscapular and suprailiac) measured with a Harpenden skinfold caliper (British Indicators Ltd., Herts, UK), using the equation of Durnin and Womersley [38]. Fat mass was calculated by multiplying body mass with the percentage of the estimated body fat, and lean mass by subtracting fat mass from body mass. Body mass index (BMI) was calculated using the following equation: BMI = Body Mass (kg)/[height (m)]^2^. BMI for age percentiles were obtained using the LMS method for boys and girls according to age [39].

#### 2.3.2. Familiarization and Standardized Warm-Up

Students were thoroughly familiarized with all the repeated sprint test and CMJ test during at least four physical education classes which took place in the month preceding the main trial. Familiarization included performance of 4–6 sprints with the same format and recovery intervals as the main test. Familiarization with CMJ measurement included 4–6 jumps per session.

The standardized warm-up performed before the main trial consisted of 5 min of light jogging and 5 min of dynamic stretching of the lower and upper body muscles [40,41,42]. Subsequently, the participants performed a specific warm-up which included running (ankling, butt kicks and high knees), jumping drills (pogo jumps and CMJ) and changes of direction (3 repetitions) [40,41,42].

#### 2.3.3. Repeated Shuttle-Sprint Running Test

The repeated shuttle-sprint running test was conducted indoors on a non-slippery court between 2.30–4.00 p.m. Five minutes after the standardized warm-up, students performed six sprints with a change of direction (180°) [16,43] at 15 m and a recovery period of 10 s in between. The time to complete each 30 m sprint with a change of direction at 15 m was measured by using a telemetric timing system (Witty, Microgate, Bolzano, Italy). Cones and tape markers were placed on the ground at the start and at 15 m when the students changed their direction. The students stood 30 cm behind the starting line, where the photogate was set, with a staggered stance [44]. The height of the photocell was 60 cm from the floor.

#### 2.3.4. Calculations

The following indices of repeated shuttle-sprint running performance were calculated:

1. **Total sprint time**: the total time of the repeated shuttle-sprint running test, calculated as the sum of the six sprint performance times [45].
2. **Percent fatigue index (FI)** was calculated from the following equation: FI = ((maximum time-minimum time))/(minimum time) × 100.
3. **Percent sprint decrement (Sdec)** was calculated from the following equation: Sdec = ((Total time)/(6 × (minimum time)))—1.
4. **Slope of the individual performance time decrement** over the six sprints was calculated using linear regression. The percentage slope decrement was calculated from the following equation: SlopeD = [((slope over 6 sprints)/(fastest time)) × 100] × 5.

**Notes**:

(a) The slope of the linear regression between sprint performance and sprint number was multiplied by 5 (i.e., number of sprints—1) to express the percent decline in performance from the first to the last sprint.
(b) The correlation coefficient of the linear regression between sprint performance and sprint number was *r* = 0.90 ± 0.08, indicating a linear decrease in sprint performance.

**Peak power during the countermovement jump (CMJ)** was estimated from the following equation [46]:

**Jump Peak Power (PP)** = 60.7 × (jump height [cm]) + 45.3 × (body mass [kg])–2055.

#### 2.3.5. Heart Rate and Blood Lactate Concentration

The participants wore heart rate monitors (Ironman easy trainer; Timex, Middlebury, CT) throughout the main trials. Heart rate was recorded 4 min after the end of the warm-up (pre) and immediately after the repeated shuttle-sprint running test (post). Blood lactate concentration was measured in a capillary blood sample taken from a fingertip 2 min after the end of the warm-up (pre) and 2 min after the repeated sprints test (post) using a portable blood lactate analyzer (Lactate Scout+, EKF Diagnostics, Cardiff, UK).

#### 2.3.6. Countermovement Jump Test

CMJ was calculated from flight time, measured by an optical system (Optojump next, Microgate, Bolzano, Italy). The students were asked to jump as high as possible with their arms on their hips (akimbo) maintaining the same body position during take-off and landing [42]. Three jumps were performed interspersed with 30 s of rest, and the best performance was used as a baseline value. CMJ was measured again one minute after the end of the repeated shuttle-sprint running test.

### 2.4. Statistical Analysis

Statistical analyses were conducted with SPSS (IBM SPSS Statistics Version 23). All data are presented as means and standard deviations (SD). A priori power analysis indicated that a total sample size of 20 participants would be needed to detect a medium effect size (Cohen’s d) of 0.50. Recent studies examining the differences between adolescent boys and girls in a repeated sprint test provide Cohen’s d values ranging from 0.44 to 0.75 [47]. Power analysis was performed using the following parameters: type of analysis was set to repeated measures mixed ANOVA (within- and between-subjects’ effects), the required power was set to 0.80, alpha was set to 0.05, and the correlation between repeated measures was set to *r* = 0.5 (G-Power software, v.3.1.9.2).

Two-way mixed ANOVA (between- and within-groups—6 sprints × 2 groups [boys and girls]) was used to examine differences between the six sprints in boys and girls. Two-way mixed ANOVAs (2 × 2) were also conducted to examine differences in CMJ height, PP, blood lactate and heart rate between pre- and post-test values in boys and girls. Moreover, two-way mixed ANOVAs (2 × 3) were conducted to examine differences in fatigue indices between boys and girls. Paired *t*-tests were also performed to examine differences in anthropometric measurements. Relationships between variables were determined by the Pearson product-moment correlation coefficient. Effect sizes were assessed using the partial eta squared (η^2^) values. Partial eta squared (η^2^) values were classified as small (0.01 to 0.059), moderate (0.06 to 0.137) and large (>0.137). For pairwise comparisons, the effect size was assessed by Cohen’s d (small: 0.2, medium: 0.5, large: 0.8). Statistical significance was set at *p* ≤ 0.05.

## 3. Results

### 3.1. Anthropometric Measurements

Paired *t*-tests showed that boys were taller (1.73 ± 0.05 m vs. 1.65 ± 0.07 m; *p* < 0.01; d = 1.47), heavier (69.1 ± 6.4 kg vs. 58.4 ± 8.4 kg; *p* < 0.01; d = 1.54), and had a greater BMI (23.0 ± 1.5 vs. 21.3 ± 2.1 kg∙m^−2^; *p* < 0.05; d = 1.01), lower percentage of body fat (14.3 ± 5.2% vs. 24.7 ± 5.0%; *p* < 0.01; d = 2.11), higher lean mass (59.2 ± 5.7 kg vs. 43.7 ± 5.1 kg; *p* < 0.01; d = 2.94) and lower fat mass (10.0 ± 3.8 kg vs. 14.7 ± 4.6 kg; *p* < 0.01; d = 1.19) compared to girls. Boys were classified in higher percentiles for BMI compared with girls (77.8 ± 12.7 vs. 60.8 ± 23.7; *p* = 0.025; d = 1.00).

Percent body fat negatively correlated with CMJ performance in boys (*r* = −0.69; *p* < 0.05) and girls (*r* = −0.70; *p* < 0.05). Percent body fat also correlated with total sprint performance in boys (*r* = 0.90; *p* < 0.01), while lean body mass negatively correlated with FI and SlopeD in boys (*r* = −0.55 and *r* = −0.51; *p* < 0.05, respectively).

### 3.2. Sprint Performance and Fatigue Indices

**Figure 1:**
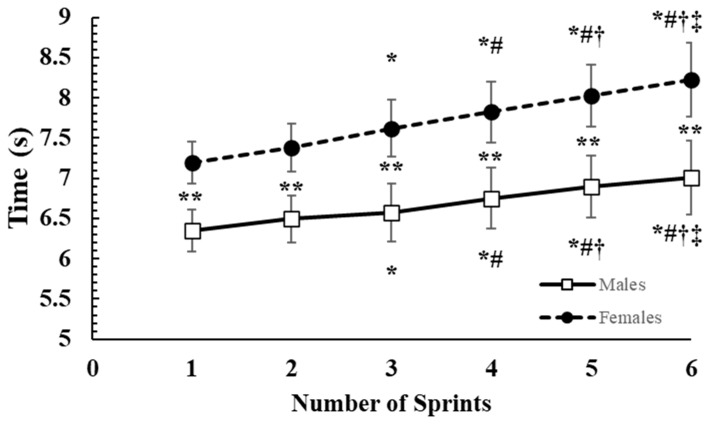
Sprint times during the repeated shuttle-sprint running test. **: *p* < 0.01 difference between boys and girls; *: *p* < 0.05 difference from 1st sprint; #: *p* < 0.05 difference from 2nd sprint; †: *p* < 0.05 difference from 3rd sprint; ‡: *p* < 0.05 difference from 4th sprint.

The two-way ANOVA showed a significant sex-by-sprint interaction effect (*p* < 0.01; η_2_ = 0.14). Tukey’s post hoc tests indicated that boys were 11.7% to 14.8% faster than girls (*p* < 0.01) in every corresponding sprint (Figure 1). Sprint time increased progressively with sprint repetitions (Figure 1). Total sprint performance time was on average 13.4% faster in boys compared with girls (40.1 ± 1.7 vs. 40.1 ± 1.7, *p* < 0.001; d = 3.62).

FI and SlopeD were almost identical and highly correlated (Slope D vs. FI: *r* = 0.97 and 0.97; SlopeD vs. Sdec: *r* = 0.86 and 0.89; *p* < 0.01 for boys and girls, respectively, see Table 1). However, Sdec was significantly lower than the two other indices (*p* = 0.001, see Table 1). Sdec also correlated with FI (*r* = 0.86 *p* < 0.01 for both boys and girls, respectively). All fatigue indices (FI, Sdec and SlopeD) were similar in boys and girls, with the *p* value of the main effect for sex approaching, but not reaching the 0.05 level (*p* = 0.06), albeit with large effect sizes (Table 1).

### 3.3. Heart Rate Responses

The two-way ANOVA did not show a group ×time interaction effect (*p* = 0.27; η^2^ = 0.05), but a significant main effect of time (*p* < 0.01; η^2^ = 0.97). Heart rate increased equally in boys and girls from pre- to post-test (*p* < 0.01; d = 6.65, Table 2) with no differences between sexes (*p* = 0.27; η^2^ = 0.05).

### 3.4. Blood Lactate Responses

Two-way ANOVA showed a significant interaction for blood lactate (*p* = 0.02; η^2^ = 0.20). Tukey’s post hoc tests revealed that blood lactate increased for both boys and girls compared with the resting values (boys: from 2.5 ± 0.6 to 14.0 ± 2.9; *p* < 0.01; d = 5.6, girls: from 2.6 ± 1.0 to 11.3 ± 3.2; *p* < 0.01; d = 3.9). However, boys had significantly higher blood lactate than girls at post-test (14.0 ± 2.9 vs. 11.3 ± 3.2; *p* = 0.02; d = 0.93) (Table 2).

### 3.5. Countermovement Jump and Jump Peak Power

There was no group × time interaction effect of CMJ performance and PP (*p* = 0.35; η^2^ = 0.04 for both variables). However, both CMJ and PP were 45.2% and 55.3% higher, respectively, for boys compared with girls (main effect of group CMJ: *p* < 0.01; η_2_ = 0.50 and PP: *p* < 0.01; η^2^ = 0.72). In addition, there was a significant main effect of time (*p* < 0.01; η^2^ = 0.73) for both CMJ and PP. CMJ decreased by 13.7 ± 7.0% (*p* < 0.01; d = 0.58) and PP decreased by 9.8 ± 5.3% (*p* < 0.01; d = 0.41) from pre- to post-test irrespective of sex (Table 1).

## 4. Discussion

The main finding of the present study was that fatigue quantified using three different indices (FI, Sdec and SlopeD) and by the drop in CMJ performance after the repeated sprints test, was similar in boys and girls despite the faster sprint times, higher CMJ and higher BL in boys. Furthermore, an interesting finding was that Sdec was almost 50% lower than the other two fatigue indices in both boys and girls, but highly correlated with the FI and SlopeD, indicating that it reflects the same quality. FI and SlopeD negatively correlated with lean body mass in boys.

Our results suggest that fatigue during repeated sprinting is similar in adolescent boys and girls. Previous studies in adults reached the same conclusion regarding the FI and Sdec during repeated sprints in men and women [28,48]. Specifically, Brooks et al. [48] found that FI during ten repeated sprints was not different between sexes, and Soydan et al. [28] reported that Sdec was similar in men and women team sport athletes performing a repeated sprint test. In contrast, several other studies found that females exhibited a lower FI or Sdec compared with males, and thus, their performance may be less influenced by fatigue during repeated sprints [27,49]. The notion that women may have higher fatigue resistance has been developed mainly from data from isometric muscle actions [50], while there are physiological mechanisms which may explain the lower fatiguability in women, such as the level of neural activation, differences in muscle metabolism and perfusion, faster metabolic recovery, as well as differences between the total mechanical work when men and women perform a similar task [51]. For example, a study in elite handball adolescent players (aged 17.7 ± 0.3 years), showed that Sdec positively correlated with 1-RM strength in the squat exercise (*r* = 0.68; *p* < 0.01) [52], linking the higher power output with higher performance decrement during a repeated sprints test [52].

In the present study, both boys and girls were not involved in any form of systematic training, and this may partially explain the similar FI, Sdec and SlopeD in boys and girls. Importantly, the tendency for girls to show greater fatigue than boys, as indicated by the large effect sizes in all fatigue indices (Table 1) and the marginally significant *p* value of the main effect of sex (*p* = 0.06), may imply a lower fitness level, which unfortunately could not be quantified in the present study. Recent data indicate that physical activity in both boys and girls declines rapidly at this age (around 15 years old), with girls being almost 40% less physically active than boys [53]. This is also true in Greece, where a large survey exhibited significantly lower physical activity in girls compared with boys [54,55]. Thus, the tendency for greater fatigue in girls may be related to decreased physical activity and the changes in body composition during adolescence, i.e., lower muscle mass and higher fat mass, which may discourage them from participating in intense physical activities or sports [56].

In this study, we found that FI, Sdec and SlopeD were strongly related with each other in both boys and girls (*r* = from 0.86 to 0.97). To our knowledge, the SlopeD derived from linear regression and expressed as a percentage of the first sprint time has not been previously used to assess fatigue in repeated sprinting. As presented in the results, the drop in performance is linear (*r* = 0.90 ± 0.08) and, unlike the FI which is calculated only from the fastest and slowest sprint, SlopeD considers performance in each sprint, thus reducing possible errors due to performance variability. SlopeD is highly correlated with FI (*r* = 0.96–0.97) and Sdec (*r* = 0.86 and 0.89), indicating that they all reflect the same quality (i.e., drop in sprint performance). As noted above, SlopeD is not only more accurate, but may also be the most sensitive, as it exhibited the highest Cohen’s d value (d = 0.89) and showed the largest difference in fatigue (10.6% vs. 14.5%) between boys and girls (Table 1). Although Sdec has been extensively used to assess fatigue during repeated sprinting and has been previously suggested to have high reliability when a large number of sprints are performed [57], more recent studies have shown that this index may be less reliable and may have lower construct validity than other measures of fatigue [57,58]. In the present study, FI and SlopeD were similar and highly correlated. However, Sdec was almost 50% lower than both FI and SlopeD (Table 1). This large discrepancy has been previously observed [58,59], but has not been thoroughly explained. Although all three fatigue indices examined in the present study highly correlated with each other, thus identifying a similar quality (i.e., fatigue), the reason why Sdec is 50% lower is that it expresses the average drop in performance throughout the repeated sprints protocol, while FI and SlopeD show the drop in performance in the last sprint. Moreover, an inherent “flaw” in the calculation of Sdec is that it takes into account a “zero” drop observed in the fastest sprint and integrates this value into the calculation of this global fatigue index, Sdec. Thus, FI and SlopeD provide the percentage drop of performance at the end of the protocol, with FI being less valid and reliable because peak performance may not be achieved in the first sprint [57]. SlopeD, on the other hand, considers all sprints in the calculation of percentage drop in performance, and thus, may be a more robust indicator of fatigue, at least in a protocol including six sprints.

It should be stressed that changes in performance time, rather than changes in the calculated external power output may be more indicative of fatigue during this protocol. For example, a recent study showed that the difference between sexes in the FI was 68.0%, when calculated based on external power which takes into account sprint performance time and body mass [Power = (body mass × 35^2^)/(time^3^)]. If we rearrange this equation to calculate the initial and final sprint times of both genders and to calculate the FI from sprint times in that study [27], then the FI is 13.6% in males and 14.7% in females, which are very close to the values observed in the present study (11.5% in boys and 15.1% in girls). Thus, the use of external power calculated from body mass and sprint performance time may provide misleading calculations regarding the drop in performance during repeated sprinting.

Another finding of the present study was that FI and SlopeD correlated with lean body mass in boys. Lean body mass and muscle strength are linked with biological maturity [60], and thus, the lower degree of fatigue observed in boys who had larger lean body mass may indicate advanced maturation among adolescent boys with the same chronological age. Notably, a recent study reported that during a repeated sprints test, FI was lower in soccer players who were born in the first quarter compared to those born in the fourth quarter of the year, suggesting a possible positive effect of advanced maturation on fatigue during sprinting [61]. Another study found that repeated-sprint performance differences among three soccer age groups disappeared when adjusted for estimated biological maturity [62]. Thus, differences in FI in the present study between boys of the same chronological age may be partially explained by differences in biological maturity.

Blood lactate after the repeated sprints test was higher in boys compared with girls (Table 2). Similar results have been obtained during laboratory repeated sprint cycling tests [28,49]. This may be explained by the differences in sprint performance times and lower limb power, as boys ran 13.4% faster and jumped 45.2% higher than girls. In addition, girls had lower lean body mass and higher fat mass compared with boys. Thus, the lower BL in girls may be due to the lower external mechanical work compared with boys [30]. Furthermore, previous studies have shown that women have higher mitochondrial density than men [63], which favors faster phosphocreatine resynthesis [64] and fat oxidation, thus reducing the reliance on glycolytic metabolism and may explain the lower BL [51,63]. The lack of a relationship between BL and fatigue during repeated sprints, also demonstrated in the present study, shows that BL may only be considered as a secondary index of sprint running performance [65].

This study has certain limitations. First, maturation was not assessed due to practical reasons. An estimate of maturation could have been useful to further interpret repeated sprint ability and fatigue. Second, due to the narrow chronological age range, the results cannot be generalized to other age groups during childhood and adolescence. Third, physical activity outside the school environment was not assessed, although it was confirmed that none of the children took part in any form of systematic organized physical activity. Last, the tendency of girls to exhibit higher fatigue indices than boys, indicated in all methods of calculation, may imply that a larger sample would reveal a difference. However, this difference would be in the opposite direction to what has been reported regarding the effects of sex on fatigue, i.e., would show a higher fatigue in girls than in boys. The possible causes of higher fatigue in girls have been discussed above and may indicate reduced fitness and physical activity level in girls at that age. Thus, the present study may stimulate further research to examine the lower ability of adolescent girls to resist fatigue during repeated high-intensity efforts, considering the increasing use of high-intensity exercise interventions for health promotion in school environments.

## 5. Conclusions

This study showed that neuromuscular fatigue during and after repeated shuttle-sprint running is similar in boys and girls, despite the faster sprint times, higher CMJ and BL in boys. Fatigue among adolescent boys of the same chronological age was related to lean body mass, indicating a possible effect of maturation on repeated sprints performance. Girls exhibited lower sprint performance, as expected, but also tended to show greater fatigue. This may be related to their possibly lower fitness level compared with boys, due to reduced physical activity at this age. Among the fatigue indices studied, FI and Slope D gave similar results, while Sdec was almost 50% lower than the other two fatigue indices in both boys and girls, as it reflects the average and not the maximum fatigue during a repeated sprints protocol. SlopeD, may be the best of the fatigue indices as it considers all sprints in the calculation of drop in performance, and thus may be a robust indicator of fatigue, at least in a protocol including six sprints.

## Figures and Tables

**Table 1:** Differences in fatigue index (FI), percent sprint decrement (Sdec), and slope of performance decrement (Slope D) during the repeated shuttle-sprint running test between boys and girls (mean ± SD). * *p* < 0.001 from FI and SlopeD.

Variables	Boys	Girls	Cohen’s d
FI (%)	11.5 ± 5.0	15.1 ± 4.3	d = 0.79
Sdec (%)	5.6 ± 2.2 *	7.3 ± 2.2 *	d = 0.80
SlopeD (%)	10.6 ± 4.8	14.5 ± 5.1	d = 0.89

**Table 2:** Heart rate, blood lactate, jump height and jump peak power before and after the repeated shuttle-sprint running test in boys and girls (mean ± SD).

Variables	Boys		Girls	
	PRE	POST	PRE	POST
Heart Rate (bpm)	93 ± 20	190 ± 12	84 ± 16	188 ± 7
Blood Lactate (mmol∙L^−1^)	2.5 ± 0.6	14.0 ± 2.9 *#	2.6 ± 1.0	11.3 ± 3.2 *
Jump Height (cm)	38.2 ± 7.3 #	33.1 ± 6.1 #	26.3 ± 4.1	22.3 ± 4.4
Jump Peak Power (W)	3392 ± 427 #	3087 ± 394 #	2184 ± 331	1944 ± 340

*: *p* < 0.01 from pre, #: *p* < 0.05 from girls in the corresponding variable and time point.

## Data Availability

The data are available upon request to the corresponding author.
